# Comparison of Linear versus Circular-Stapled Gastroenterostomy in Roux-en-Y Gastric Bypass: A Nationwide Population-Based Cohort Study

**DOI:** 10.1007/s11695-021-05436-4

**Published:** 2021-04-27

**Authors:** Marleen M. Romeijn, Stijn van Hoef, Loes Janssen, Kelly G. H. van de Pas, François M. H. van Dielen, Arijan A. P. M. Luijten, Kevin W. A. Göttgens, Jan Willem M. Greve, Wouter K. G. Leclercq

**Affiliations:** 1 Department of Surgery, Máxima Medical Center, Veldhoven, The Netherlands; 2 Research School NUTRIM, Department of Surgery, Maastricht University Medical Center, Maastricht, the Netherlands; 3 Department of Surgery, Zuyderland Medical Center, Heerlen, the Netherlands

**Keywords:** Bariatric surgery, Roux-en-Y gastric bypass, Stapled gastroenterostomy, Non-response, Weight regain

## Abstract

**Background:**

When performing a Roux-en-Y gastric bypass (RYGB), the gastroenterostomy can be constructed with a circular stapled or linear stapled technique. The size of the gastroenterostomy depends on the stapling method and this may affect weight loss outcomes. The aim of this study was to examine the impact of the stapling technique on weight loss outcomes after RYGB.

**Methods:**

This is a nationwide population-based cohort study of patients that received a RYGB. Data were derived from the Dutch Audit of Treatment of Obesity. Primary outcome was the impact of stapling technique on the rate of non-response defined as significant weight regain (≥20% of a patients’ lost weight) 2–4 years post-surgery, after initial successful weight loss (≥20% total weight loss, TWL). Secondary outcomes were the rate of response, defined as successful weight loss (≥20% TWL) within 1.5 years post-surgery, the incidence of complications and the progression of comorbidities.

**Results:**

In a cohort of 12,468 patients, non-response was equally distributed between both groups (circular 18.0% vs. linear 17.6%). No differences in response rate (circular 97.0% vs. linear 96.5%) or %TWL were observed up to 4 years post-surgery. Patients in the circular stapled group experienced more complications, specifically major bleedings (2.4% vs. 1.2%; p=0.002) within 30 days postoperatively. No differences were found in deteriorated comorbidities, neither in de novo developed comorbidities.

**Conclusion:**

When comparing stapling technique in RYGB, weight loss outcomes did not differ during a 4-year follow-up period. The linear stapled gastroenterostomy could pose an advantage due to its lower complication rate.

**Graphical abstract:**

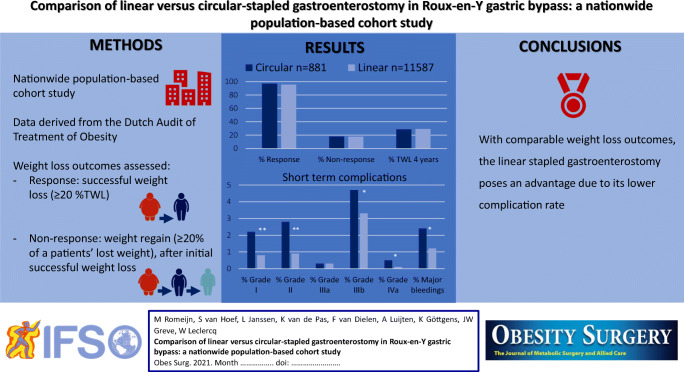

**Supplementary Information:**

The online version contains supplementary material available at 10.1007/s11695-021-05436-4.

## Introduction

Bariatric surgery is considered the best option for sustained weight loss in morbidly obese patients [1, 2]. The laparoscopic Roux-en-Y gastric bypass (RYGB) is the most commonly performed primary bariatric procedure in the Netherlands [3]. Within the last 5 years, approximately 58,000 bariatric procedures have been performed including RYGB surgery in 59–75% [4]. During the creation of the RYGB, the gastroenterostomy can be constructed in three different ways: circular stapled, linear stapled, or completely hand-sewn. Worldwide, there is a large variety in applied techniques because to date, no surgical technique has been superior to the other [5]. Compared to the two stapling techniques, hand sewing is less frequently performed because it is technically demanding and not reproducible [5]. An important difference between the two stapling techniques is anastomotic size. Where the circular stapled anastomosis (CSA) usually has a diameter between 21 and 25 mm depending on the device used, the diameter of the linear stapled anastomosis (LSA) is assumed to be wider with a diameter between 20 and 45 mm [6, 7]. Besides this, there is a financial difference as the circular stapling technique is more expensive.

It is known that 25–35% of patients after RYGB do not achieve adequate weight loss, or regain an excessive amount of weight after initial adequate weight loss [8–10]. This can be related to lifestyle, hormonal, and metabolic factors, but may also be explained by surgical factors like an enlarged pouch or gastroenterostomy [11–13]. A wide gastroenterostomy has been defined as exceeding 2 cm [11] and forms the basis of many currently used treatment strategies. These strategies aim to correct the size of the anastomosis through sclerotherapy, argon plasma coagulation, endoscopic plication, and endoscopic suturing [13, 14].

As the size of the initial gastroenterostomy depends on the stapling technique (CSA versus LSA), one may reason that the stapling technique could influence weight loss outcome. Based on a nationwide study performed in Sweden, no differences in excess body mass index (BMI) loss nor total weight loss (TWL) were found 1 year after RYGB when comparing CSA with LSA [15]. Bohdjalian et al. found no differences in excess weight loss 1 and 2 years after RYGB when comparing the two techniques [6] and furthermore, Langer et al. found no differences in excess BMI loss up to 5 years after RYGB [16]. Both studies were designed as a matched-pair study and included only 150 patients.

To date, research has not yet described the impact of stapling technique on mid-term weight loss outcomes and importantly on weight regain in a high volume of patients. Therefore, the aim of this nationwide study was to assess the impact of stapling technique in RYGB on weight loss outcomes including weight regain (i.e., non-response) in a follow-up period of 4 years.

## Method

### Study Population

This is a nationwide, population-based cohort study of patients that received a RYGB in the Netherlands. A pseudonymized dataset was obtained from the Dutch Audit of Treatment of Obesity (DATO), a registry covering all bariatric procedures performed within the Netherlands since 1 January 2015. Details on this registry and the recorded variables have been published before [3]. Patients were included if they underwent primary RYGB, between the age of 18 and 65 years, with a BMI ≥40.0 kg/m^2^ or ≥35.0 kg/m^2^, and suffering from an obesity-related comorbidity. The RYGB had taken place between 1 January 2015 and 31 December 2017. Eligibility for surgery was confirmed after evaluation by a multidisciplinary team and was in accordance with the International Federation for the Surgery of Obesity and Metabolic Disorders (IFSO) guidelines [17]. Follow-up weights should be noted within 1.5 years and at 2 years for inclusion. Exclusion criteria were hand-sewn gastroenterostomy, a bariatric procedure other than RYGB (such as one-anastomosis gastric bypass or banded bypass), and revisional or secondary procedures.

### Study Outcomes

The primary outcome of this study is the rate of non-response defined as significant weight regain (≥20% of a patients’ lost weight) 2–4 years post-surgery, after initial successful weight loss (≥20% TWL). The threshold of 20% weight regain is based on the study by Uittenbogaart et al., whereas the threshold of 20% TWL is based on the DATO registry and previous publications [4, 18, 19]. Secondary outcomes include the rate of response defined as successful weight loss (≥20% TWL) within 1.5 years after RYGB, weight loss expressed in both TWL and change in BMI, the incidence of complications, and the progression of obesity-related comorbidities. The percentage of TWL was calculated as (preoperative weight − follow-up weight)/(preoperative weight) × 100%. In addition, the change in BMI was calculated as (preoperative BMI − follow-up BMI).

Obesity-related comorbidities included type 2 diabetes mellitus (DM), hypertension, hyperlipidemia, gastroesophageal reflux disease (GERD), obstructive sleep apnea syndrome (OSAS), and osteoarthritis. The definition of these comorbidities is based on the ASMBS guideline by Brethauer et al. [3, 4, 20]. Comorbidities were recorded regardless of an active treatment. The comorbidities were categorized as resolved, improved, unchanged, deteriorated, and de novo. Because the status of the comorbidity at 3 and 4 years postoperatively was frequently missing, this outcome was assessed up to 2 years after surgery. Postoperative complications were registered both on short term (i.e., <30 days) and long term, and were categorized according to the Clavien–Dindo Classification of Surgical Complications (CD) [21]. A severe complication was defined as CD grade IIIb (i.e., complication requiring intervention under general anesthesia) or higher. Mortality was recorded as CD grade V and included death from a postoperative complication.

### Surgical Technique

The CSA was performed in a standardized fashion by four high-volume surgeons located in two centers. This stapling technique was previously described in detail by Dillemans et al. [22]. The technique involves introduction of a circular stapler of 25 mm through a left lateral abdominal port site (2–3 cm). The anvil of the stapler is inserted into an opening in the gastric pouch and secured with a purse string suture. The biliopancreatic (BP) limb is then opened over a 2–3 cm length to introduce the stapler. After connecting the anvil with the stapler, the anastomosis is created. At the BP side of the anastomosis, the small intestine is closed and cut with a linear stapler to divide the limbs. The LSA was performed as standardized fashion by 15 surgeons located in 18 centers. This technique was published as an original technique in 2003 [23]. A small opening is made in the alimentary (AL) limb to introduce one side of the linear stapler, which is then inserted into a small opening in the gastric pouch with its other side. After firing and removing the stapler, the small opening through which the stapler was introduced is closed using a resolvable suture or with another stapler. At the BP side of the anastomosis, the small intestine is closed and cut with a linear stapler to divide the limbs. No intestine has to be excised with this technique.In both stapling techniques, the limb lengths were either estimated or measured prior to construction. Both techniques provide the option of closure of the mesenteric and Petersen’s defects in order to limit the risk of internal hernias.

### Statistical Analyses

Statistical analyses were performed using IBM SPSS statistics software, version 22.0. A *p* value of <0.05 was considered statistically significant. Continuous variables are presented as mean ± SD, while categorical variables are presented as absolute number (percentage). Categorical variables were compared with the *χ*^2^ test, and continuous variables with an independent *t* test. The association between non-response rates (outcome) and stapling technique (exposure) is analyzed using multivariate logistic regression. Within these analyses, corrections were made for known confounders based on literature (baseline BMI, age at surgery, gender [24]) and variables that may have a confounding effect based on univariate analysis (variables that are associated with the outcome with a *p* value <0.1 in a univariate analysis). Stratification was applied to explore effect modification by gender and age at surgery which was statistically tested by including an interaction variable into the regression model. Sensitivity analyses were performed to test the robustness of the findings to missing data or possible variation in definitions and classifications.

## Results

**Fig. 1:**
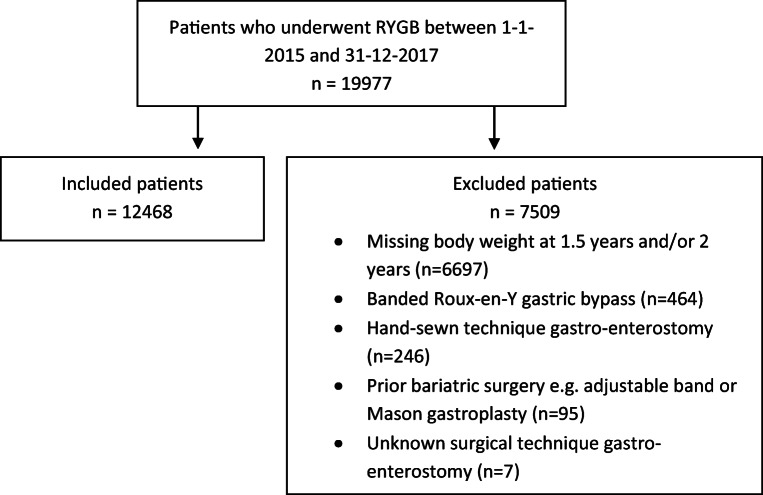
Flow diagram of inclusion and exclusion of patients

A total of 19,977 patients were registered during the study period (Fig. 1). A significant number of patients were excluded due to missing values in essential variables at various time points. In total, 12,468 patients were included in the study, 881 in the CSA group and 11,587 in the LSA group. In the CSA group, 881 patients (100.0%) completed ≤1.5 and 2 years of follow-up, 444 patients (50.4%) completed 3 years of follow-up, and 186 patients (21.1%) completed 4 years of follow-up. In the LSA group, 11,587 patients (100.0%) completed ≤1.5 and 2 years of follow-up, 6235 patients (53.8%) completed 3 years of follow-up, and 2694 patients (23.3%) completed 4 years of follow-up.

As shown in Table 1, preoperative BMI was equally distributed between the CSA and the LSA group (42.7 kg/m^2^ vs. 42.8 kg/m^2^, respectively). The CSA group had statistically significant lower numbers of preoperative type 2 DM, GERD, and osteoarthritis compared to the LSA group. Furthermore, the CSA group suffered from more short-term complications (CD grade I, CD grade II, CD grade IIIb, and CD grade IVa) than the LSA group (all *p*≤0.05). There were significantly more postoperative major bleedings in the CSA group (2.4% vs. 1.2%, *p*=0.002). In the long term, the CSA group suffered from more gallstones, incisional hernias, bowel obstructions, and internal hernias (Supplementary Table 1). In the CSA group, the most common length of the BP limb was 70 cm (57.0%) and 150 cm for the AL limb (80.2%). In the LSA group the length of the BP limb largely varied (65.6%, 50–80cm; 26.5%, 150 cm), while the most common length of the AL limb was 150 cm (60.8%).

**Table 1:** Baseline characteristics of the study population

Gender, no. (%)			
Female	713 (80.9)	9541 (82.3)	0.291
Age (years)	45.2 ±10.5	45.1±10.6	0.885
Preoperative comorbidities, no. (%)			
Hypertension	333 (37.8)	4269 (36.8)	0.571
Type II diabetes mellitus	143 (16.2)	2595 (22.4)	<0.001*
Hyperlipidemia	169 (19.2)	2523 (21.8)	0.071
Gastroesophageal reflux disease	89 (10.1)	1667 (14.4)	<0.001*
OSAS	265 (30.1)	2231 (19.3)	<0.001*
Osteoarthritis	344 (39.0)	5699 (49.2)	<0.001*
Preoperative weight (kg, ±SD)	122.7±18.6	122.7±17.9	0.994
Preoperative BMI (kg/m^2^ ±SD)	42.7±4.9	42.8±4.7	0.265
Laparoscopic, no. (%)	879 (99.8)	11573 (99.9)	0.396
Length of biliopancreatic limb (cm ±SD)	72.9±15.3	90.5±38.6	<0.001*
Length of alimentary limb (cm ±SD)	145.8±9.7	133.6±33.7	<0.001*
Length of hospital stay (days ±SD)	2.3±1.7	1.5±2.7	<0.001*
Number of readmissions (<30 days), no. (%)	25 (2.8)	283 (2.4)	0.466
Postoperative complication <30 days, no. (%)			
CD grade I	19 (2.2)	89 (0.8)	<0.001*
CD grade II	25 (2.8)	99 (0.9)	<0.001*
CD grade IIIa	3 (0.3)	29 (0.3)	0.610
CD grade IIIb	41 (4.7)	382 (3.3)	0.032*
CD grade IVa	4 (0.5)	15 (0.1)	0.017*
CD grade IVb	–	9 (0.1)	0.408
CD grade V	–	1 (0.0)	0.783
Type of complication, no. (%)			
Major bleeding	21 (2.4)	136 (1.2)	0.002*
Anastomotic leakage	1 (0.1)	43 (0.4)	0.214
Intra-abdominal abscess	2 (0.2)	13 (0.1)	0.343
Wound infection	1 (0.1)	14 (0.1)	0.952
Intestinal obstruction	1 (0.1)	28 (0.2)	0.447
Anastomotic stricture	0 (0.0)	1 (0.0)	0.783

Data presented as number (%) or mean (SD). *p value is below the threshold of <0.05

CD = Clavien–Dindo classification, CSA = circular-stapled anastomosis, LSA = linear-stapled anastomosis, OSAS = obstructive sleep apnea syndrome, BMI = body mass index. IIIa is a complication requiring intervention under local anesthesia; IIIb is a complication requiring general anesthesia; IVa is a complication resulting in single organ failure; IVb is a complication resulting in multiple organ failure; V is a complication resulting in death

When using ≥20% TWL as threshold for response (i.e., successful weight loss), there were no significant differences between the groups (Table 2). Based on the aforementioned criteria for non-response (i.e., weight regain after successful weight loss), there were also no significant differences. The percentage of TWL was similar in the CSA and LSA group, with a mean of 28.6% and 29.1% 4 years after surgery (*p*=0.533).

**Table 2:** Weight loss outcomes comparing circular- and linear-stapled anastomosis of the gastroenterostomy

	CSA		LSA		
	No. in analysis^3^	No. (%)	No. in analysis^3^	No. (%)	*p* value
Response rate^1^	881	855 (97.0)	11,587	11,177 (96.5)	0.360
Non-response rate^2^	881	159 (18.0)	11,587	2045 (17.6)	0.765
	No.	% ±SD	No.	% ±SD	*p* value
TWL based on lowest weight within 1.5 years	881	33.9 ±7.5	11,587	33.4 ±7.6	0.046*
TWL 1.5 years	655	33.5 ±7.9	6812	33.7 ±8.0	0.416
TWL 2 years	881	32.9 ±8.3	11,587	32.7 ±8.5	0.613
TWL 3 years	444	30.9 ±8.2	6235	31.0 ±9.0	0.884
TWL 4 years	186	28.6 ±8.7	2694	29.1 ±10.1	0.533
	No.	% ±SD	No.	% ±SD	*p* value
∆Change in BMI based on lowest weight within 1.5 years	881	14.6 ±4.0	11,587	14.4 ±3.8	0.142
∆Change in BMI 1.5 years	655	1434 ±4.2	6812	14.5 ±4.0	0.386
∆Change in BMI 2 years	881	14.2 ±4.4	11,587	14.1 ±4.2	0.781
∆Change in BMI 3 years	444	13.4 ±4.6	6235	13.4 ±4.4	0.909
∆Change in BMI 4 years	186	12.4 ±4.9	2694	12.6 ±4.7	0.629

Data presented as number (%) or mean (SD). *p value is below the threshold of <0.05

CSA = circular-stapled anastomosis, LSA = linear-stapled anastomosis, TWL = total weight loss

^1^Defined as successful weight loss (≥20% total body weight loss) within 1.5 years after surgery

^2^Defined as significant weight regain (≥20% of a patients’ lost weight) after initial successful weight loss (≥20% total body weight loss) 2 years after surgery

^3^The total amount of patients included in the CSA group was 881 patients. The total amount of patients included in the LSA group was 11,587 patients

Table 3 displays the results of the univariate and multivariate analyses indicating which variables are associated with non-response. Univariate analysis revealed that stapling technique was not associated with non-response (OR 1.03; 95% CI 0.86–1.23). Based on the multivariate analysis, a male gender and preoperative hypertension was associated with an increased risk of non-response (OR 1.29 and OR 1.16, respectively). Contrary, preoperative type 2 DM, preoperative GERD, a higher age and a longer BP limb were associated with a decreased risk of non-response (OR 0.78, OR 0.70, OR 0.99, and OR 0.99, respectively). When we included the interaction variables gender and age in the model, the ORs changed only slightly without affecting the abovementioned findings. Interestingly, the length of the AL limb and the other comorbidities (hyperlipidemia, OSAS, osteoarthritis) were not associated with non-response.

**Table 3:** Results of univariate (unadjusted OR) and multivariate (adjusted OR) logistic regression of variables associated with non-response after RYGB

	Unadjusted OR	95% CI	*p* value	Adjusted OR	95% CI	*p* value
Stapling technique (circular)	1.03	0.86–1.23	0.765	0.93	0.78–1.12	0.439
Gender (male)	1.34	1.20–1.50	<0.001^#^	1.29	1.14–1.47	<0.001*
Age at surgery (years)	0.99	0.99–1.00	0.001^#^	0.99	0.98–0.99	<0.001*
Preoperative BMI (kg/m^2^)	0.99	0.98–1.00	0.253			
Preoperative hypertension (yes)	1.11	1.01–1.21	0.032^#^	1.16	1.03–1.29	0.014*
Preoperative type 2 DM (yes)	0.97	0.95–0.98	<0.001^#^	0.78	0.69–0.89	<0.001*
Preoperative hyperlipidemic (yes)	1.01	0.91–1.13	0.830			
Preoperative GERD (yes)	0.68	0.58–0.78	<0.001^#^	0.70	0.60–0.82	<0.001*
Preoperative OSAS (yes)	1.12	1.00–1.25	0.044^#^	1.01	0.88–1.15	0.868
Preoperative osteoarthritis (yes)	0.91	0.83–0.99	0.035^#^	0.92	0.83–1.02	0.105
Length of biliopancreatic limb (cm)	0.99	0.99–1.00	<0.001^#^	0.99	0.99–1.00	<0.001*
Length of alimentary limb (cm)	1.00	1.00–1.00	<0.001^#^	1.00	1.00–1.00	0.306
Length of hospital stay (days)	1.01	1.00–1.02	0.156			
Complication by Clavien–Dindo classification (yes)	1.09	0.95–1.27	0.204			

Dependent variable: % non-response. #*p* value is below the threshold of <0.1; therefore, this variable is included in the multivariate analysis. *p value is below the threshold of <0.05

DM = diabetes mellitus, GERD = gastroesophageal reflux disease, OSAS = obstructive sleep apnea syndrome

Table 4 displays the effect of stapling technique on the progression of obesity-related comorbidities. There was no significant difference in deterioration of comorbidities in the CSA compared to the LSA group, neither in de novo developed comorbidities. In the CSA group, there was a better resolution of hypertension and OSAS (65.1% vs. 52.8%; 76.3% vs. 64.3%). Instead in the LSA group, these comorbidities were more often merely improved (25.6% vs. 13.0%; 19.4% vs. 7.5%). Moreover, in the LSA group there was a better resolution of GERD and osteoarthritis than in the CSA group (75.2% vs. 62.3%; 43.8% vs. 36.8%).

**Table 4:** Progression of obesity-related comorbidities between circular- and linear-stapled anastomosis of the gastroenterostomy

	CSA 1.5–2 years of follow-up						LSA 1.5–2 years of follow-up					
	No.^1^	Resolved no. (%)^2^	Improved no. (%)^2^	Unchanged no. (%)^2^	Deteriorated no. (%)^2^	De novo no. (%)^3^	No.^1^	Resolved no. (%)^2^	Improved no. (%)^2^	Unchanged no. (%)^2^	Deteriorated no. (%)^2^	De novo no. (%)^3^
Hypertension	324/333	65.1**	13.0**	21.6	0.3	7.1	3258/4269	52.8**	25.6**	20.3	1.1	2.0
Type 2 DM	133/143	69.2	26.3	3.8	0.8	0.0	1706/2595	69.9	21.5	7.6	0.9	0.0
Hyperlipidemia	76/169	51.3	13.2	35.5	0.0	0.0	1399/2523	55.8	16.2	27.4	0.6	3.6
OSAS	253/265	76.3**	7.5**	16.2	0.0	0.0	1394/2231	64.3**	19.4**	15.7	0.5	5.8
GERD	53/89	62.3*	17.0	20.8	0.0	0.7	499/1667	75.2*	11.0	12.6	0.8	3.1
Osteoarthritis	280/344	36.8*	32.9*	28.2	2.1	1.9	2458/5699	43.8*	26.7*	24.3	4.5	2.2

^1^Number of patients from which comorbidity status is known at 1.5–2 years of follow-up/number of patients with preoperative comorbidity

^2^Based on the number of patients with preoperative comorbidity and known status at 1.5–2 years of follow-up (numerator in “No.” column)

^3^Based on the number of patients without preoperative comorbidity and known status at 1.5–2 years of follow-up

*p value is below the threshold of 0.05, **p value is below the threshold of 0.01

CSA = circular-stapled anastomosis, DM = diabetes mellitus, GERD = gastroesophageal reflux disease, OSAS = obstructive sleep apnea syndrome, LSA = linear-stapled anastomosis

## Discussion

Preoperative knowledge on factors related to insufficient weight loss and weight regain after bariatric surgery is crucial. Within this topic, lifestyle, hormonal, and surgical factors have been an area of great interest [8, 9, 13]. It was hypothesized that the stapling technique used in RYGB construction may contribute to non-response, as it affects the size of the gastroenterostomy and an enlarged anastomosis size is associated with weight regain [11–13]. In this study reporting on 12,468 patients, it was shown that surgical technique (CSA vs. LSA) does not affect non-response rate nor TWL up to 4 years after RYGB. The results regarding TWL were similar to those reported by other authors [25, 26].

As no difference in weight loss outcomes was found, it can be suggested that the diameter of the gastroenterostomy may not be of influence. Caution must be applied here, as it can only be speculated what the actual diameter of the anastomosis was and how this varied between the two stapling techniques. Technical information about the anastomotic diameter is not available and controversy is still present regarding which measurement gives the most reliable assessment. Besides this, if we would assume that a larger anastomotic diameter allows more passage of food and less satiety, there must be another explanation. For instance, a larger anastomotic diameter could more easily cause dumping and this may, in turn, have a restraining effect as patients want to prevent these dumpings. This explanation could play a role in the balance between anastomotic diameter and caloric intake.

The number of patients in the CSA group that experienced a short-term complication, specifically CD grades I, II, IIIb, and IVa, was higher than expected. Based on previous reports, the rate of these complications is estimated at 0.5–1.5%, 0.2–1.3%, 1.9%, and 0.7% for CD grades I, II, IIIb, and IVa, respectively [4]. The patients in the CSA group experienced more postoperative major bleedings with an average of 2.4%, being nearly twice as high as the national average [27]. Yet, this finding is in line with prior literature [15, 28–30]. The origin (i.e., intraperitoneal or intraluminal) of bleedings reported in this study as well as the need for interventions were unfortunately unknown. Nevertheless, it is likely that these bleedings accounted for the CD grade IIIb–IVa complications and thus resulted in relaparoscopy with general anesthesia and/or single-organ dysfunction [21]. Possible explanations for finding more bleedings in the CSA group are differences in stapler height, the number of stapler rows, and reinforcement of the staple line [15, 28–30]. This could also be influenced by local differences in routine drain placement, hemoglobin testing, and thromboembolic prophylaxis. Continued efforts are needed to lower the incidence of bleedings in particular when performing a circular stapled RYGB.

In order to identify patients that are at risk of developing non-response, multiple factors have been investigated and so far, a pattern of an older age, a higher preoperative BMI, the presence of comorbidities, and behavioral and psychosocial factors have been shown to predict non-response [8, 24, 31–33]. The current study showed male gender and preoperative hypertension increased the risk of non-response. This finding supports the work of other studies [32, 33], although conflicting results were also found [24]. One remarkable finding was that preoperative type 2 DM and GERD lowered the risk of non-response. This is in contrast to the study by Stenberg et al. who found that preoperative DM was associated with a reduced %TWL after 5 years, although GERD was not associated with less %TWL [32]. There is no clear explanation for this controversy although hypothetically, patients with type 2 DM might be better motivated to keep their weight off in order to prevent recurrence of their disease and resumption of therapy. The insights gained from this study may contribute to a broader understanding of the characteristics of patients that develop non-response.

The current study showed that hypertension and OSAS had a better resolution in the CSA group. The resolution of hypertension is consistent with other studies, while the resolution of OSAS was strikingly high [34]. Notably, a high percentage of OSAS was observed preoperatively in the CSA group (CSA 30.1% vs. LSA 19.3%). The reason why the circular stapled technique was superior in the resolution of this comorbidity cannot easily be explained and is not in line with (limited) available literature [35]. A hypothesis may be that resolution or improvement of comorbidities has been assessed, interpreted, and registered differently in the centers. Another hypothesis is that the sample size was too low, resulting in a type II error. These factors may have led to erroneous conclusions and may be responsible for the contrasting findings of this study.

There are other aspects that should be considered when comparing the LSA with the CSA. Previous studies showed that the LSA reduces costs (£824 for materials per patient reported by Fehervari et al.; 250USD for used staplers per patient reported by Major et al.), operation time, as well as length of hospital stay [15, 29, 36, 37]. However, there are no studies that have assessed whether the LSA results in less postoperative pain and thus earlier mobilization. The rationale behind this could be that the larger left lateral incision, to allow access of the circular stapler, causes more pain due to more muscle/nerve damage during dissection. As a next step, comparative studies should be designed focusing on pain and mobilization, but also on broader clinical outcomes like quality of life and treatment satisfaction.

This study presents three limitations. First, as data from multiple centers were included in this study, there may have been differences in protocols that influenced weight loss outcomes. The total duration, frequency, and adherence to follow-up appointments within the Dutch centers varies greatly, possibly effecting the development and signaling of weight regain [38]. Second, the average lost to follow-up 4 years after primary surgery in the Dutch centers is approximately 52% and this may be an important source of selection bias, as weight regain could be a reason for not showing up [39]. Third, the retrospective nature of this study may have accounted for errors in data entry, miscoding, and interpretation. Despite these limitations, this study is the first nationwide cohort study reporting mid-term weight loss outcomes and in particular non-response rates in LSA and CSA. Taking the incidence of complications, weight loss outcomes and reported costs into account, the LSA presents an advantage and could be favored.

## Conclusion

In this comparative study reporting on 12,468 patients, it is demonstrated that the surgical technique used during gastroenterostomy construction (circular vs. linear) in RYGB does not affect weight loss, nor does it affect the risk of weight regain. The percentage of postoperative complications, particularly major bleedings within 30 days, was significantly higher in the circular stapled technique (2.4% vs. 1.2%). Based on this, the linear stapled technique could be favored. No differences were found in deteriorated comorbidities and neither in de novo developed comorbidities between the two techniques. One unanticipated finding was that the circular stapling technique resulted in a better resolution of hypertension and OSAS, although these results should be interpreted with caution as it can be debated whether this study had sufficient power to assess these outcomes. A further study should be designed, preferably a randomized controlled trial, with extensive follow-up rate to definitively demonstrate superiority of one of these stapling techniques.

## Supplementary Information

ESM 1 (DOCX 13 kb)

## Abbreviations

AL: alimentary limb
BMI: body mass index
BP: biliopancreatic limb
CD: Clavien–Dindo
CSA: circular stapled anastomosis
DATO: Dutch Audit of Treatment of Obesity
DM: diabetes mellitus
GERD: gastroesophageal reflux disease
LSA: linear stapled anastomosis
OSAS: obstructive sleep apnea syndrome
RYGB: Roux-en-Y gastric bypass
TWL: total weight loss

## References

[1] Buchwald H, Avidor Y, Braunwald E, Jensen MD, Pories W, Fahrbach K, Schoelles K (2004). Bariatric surgery: a systematic review and meta-analysis. J Am Med Assoc.

[2] Puzziferri N, Roshek TB, Mayo HG, Gallagher R, Belle SH, Livingston EH (2014). Long-term follow-up after bariatric surgery: a systematic review. JAMA.

[3] Poelemeijer Y, Liem R, Nienhuijs S (2018). A Dutch Nationwide Bariatric Quality Registry: DATO. Obes Surg.

[4] Jaarrapportage 2019 DATO [Internet]. Dutch Institute for Clinical Auditing. 2019. Available from: https://dica.nl/jaarrapportage-2019/dato.

[5] Kumar P, Yau HV, Trivedi A, Yong D, Mahawar K (2020). Global variations in practices concerning Roux-en-Y gastric bypass—an online survey of 651 bariatric and metabolic surgeons with cumulative experience of 158,335 procedures. Obes Surg.

[6] Bohdjalian A, Langer FB, Kranner A, Shakeri-Leidenmühler S, Zacherl J, Prager G (2010). Circular- vs. linear-stapled gastrojejunostomy in laparoscopic Roux-en-Y gastric bypass. Obes Surg.

[7] Penna M, Markar SR, Venkat-Raman V, Karthikesalingam A, Hashemi M (2012). Linear-stapled versus circular-stapled laparoscopic gastrojejunal anastomosis in morbid obesity: meta-analysis. Surg Laparosc Endosc Percutan Tech.

[8] Cooper TC, Simmons EB, Webb K, Burns JL, Kushner RF (2015). Trends in weight regain following Roux-en-Y gastric bypass (RYGB) bariatric surgery. Obes Surg.

[9] Amundsen T, Strømmen M, Martins C (2017). Suboptimal weight loss and weight regain after gastric bypass surgery-postoperative status of energy intake, eating behavior, physical activity, and psychometrics. Obes Surg.

[10] Uittenbogaart M, de Witte E, Romeijn M, Luijten A, van Dielen F, Leclercq W (2020). Primary and secondary nonresponse following bariatric surgery: a survey study in current bariatric practice in the Netherlands and Belgium. Obes Surg.

[11] Heneghan HM, Yimcharoen P, Brethauer SA, Kroh M, Chand B (2012). Influence of pouch and stoma size on weight loss after gastric bypass. Surg Obes Relat Dis.

[12] Abu Dayyeh BK, Lautz DB, Thompson CC (2011). Gastrojejunal stoma diameter predicts weight regain after Roux-en-Y gastric bypass. Clin Gastroenterol Hepatol.

[13] Maleckas A, Gudaitytė R, Petereit R, Venclauskas L, Veličkienė D (2016). Weight regain after gastric bypass: etiology and treatment options. Gland Surg.

[14] Storm AC, Thompson CC (2017). Endoscopic treatments following bariatric surgery. Gastrointest Endosc Clin N Am.

[15] Edholm D, Sundbom M (2015). Comparison between circular- and linear-stapled gastrojejunostomy in laparoscopic Roux-en-Y gastric bypass—a cohort from the Scandinavian Obesity Registry. Surg Obes Relat Dis.

[16] Langer FB, Prager G, Poglitsch M, Kefurt R, Shakeri-Leidenmühler S, Ludvik B, Schindler K, Bohdjalian A (2013). Weight loss and weight regain-5-year follow-up for circular- vs. linear-stapled gastrojejunostomy in laparoscopic Roux-en-Y gastric bypass. Obes Surg.

[17] Fried M, Yumuk V, Oppert J, Scopinaro N, Torres A, Weiner R (2014). Interdisciplinary European guidelines on metabolic and bariatric surgery. Obes Surg.

[18] Uittenbogaart M, Leclercq W, Luijten A, Romeijn M, Bonouvrie D, van Dielen F (2019). Defining an international standard for primary and secondary non-response following bariatric surgery for research purposes: a modified Delphi consensus. Surg Obes Relat Dis.

[19] Corcelles R, Boules M, Froylich D, Hag A, Daigle CR, Aminian A, Brethauer SA, Burguera B, Schauer PR (2016). Total weight loss as the outcome measure of choice after Roux-en-Y gastric bypass. Obes Surg.

[20] Brethauer SA, Kim J, el Chaar M, Papasavas P, Eisenberg D, Rogers A, Ballem N, Kligman M, Kothari S, ASMBS Clinical Issues Committee (2015). Standardized outcomes reporting in metabolic and bariatric surgery. Surg Obes Relat Dis.

[21] Dindo D, Demartines N, Clavien PA (2004). Classification of surgical complications: a new proposal with evaluation in a cohort of 6336 patients and results of a survey. Ann Surg.

[22] Sakran N, Assalia A, Sternberg A, Kluger Y, Troitsa A, Dillemans B (2011). Smaller staple height for circular stapled gastrojejunostomy in laparoscopic gastric bypass: early results in 1,074 morbidly obese patients. Obes Surg.

[23] Olbers T, Lönroth H, Fagevik-Olsén M, Lundell L (2003). Laparoscopic gastric bypass: development of technique, respiratory function, and long-term outcome. Obes Surg.

[24] Shantavasinkul PC, Omotosho P, Corsino L, Portenier D, Torquati A (2016). Predictors of weight regain in patients who underwent Roux-en-Y gastric bypass surgery. Surg Obes Relat Dis.

[25] Sjöström L (2013). The Sahlgrenska Academy, The University of Gothenburg, Gothenburg, Sweden Review of the key results from the Swedish Obese Subjects (SOS) trial – a prospective controlled intervention study of bariatric surgery (review). J Intern Med.

[26] Arterburn D, Wellman R, Emiliano A, Smith SR, Odegaard AO, Murali S, Williams N, Coleman KJ, Courcoulas A, Coley RY, Anau J, Pardee R, Toh S, Janning C, Cook A, Sturtevant J, Horgan C, McTigue KM, for the PCORnet Bariatric Study Collaborative (2018). Comparative effectiveness and safety of bariatric procedures for weight loss: a PCORnet cohort study. Ann Intern Med.

[27] Poelemeijer YQM, Liem RSL, Våge V, Mala T, Sundbom M, Ottosson J, Nienhuijs SW (2018). Perioperative outcomes of primary bariatric surgery in North-Western Europe: a pooled multinational registry analysis. Obes Surg.

[28] Finks JF, Carlin A, Share D, O’Reilly A, Fan Z, Birkmeyer J, Birkmeyer N (2011). Michigan Bariatric Surgery Collaborative from the Michigan Surgical Collaborative for Outcomes Research Evaluation. Effect of surgical techniques on clinical outcomes after laparoscopic gastric bypass—results from the Michigan Bariatric Surgery Collaborative. Surg Obes Relat Dis.

[29] Major P, Janik MR, Wysocki M, Walędziak M, Pędziwiatr M, Kowalewski PK, Małczak P, Paśnik K, Budzyński A (2017). Comparison of circular- and linear-stapled gastrojejunostomy in laparoscopic Roux-en-Y gastric bypass: a multicenter study. Wideochir Inne Tech Maloinwazyjne.

[30] Jiang HP, Lin LL, Jiang X, Qiao HQ (2016). Meta-analysis of hand-sewn versus mechanical gastrojejunal anastomosis during laparoscopic Roux-en-Y gastric bypass for morbid obesity. Int J Surg.

[31] Hindle A, de la Piedad Garcia X, Brennan L (2017). Early post-operative psychosocial and weight predictors of later outcome in bariatric surgery: a systematic literature review. Obes Rev.

[32] Stenberg E, Näslund I, Persson C, Szabo E, Sundbom M, Ottosson J, Näslund E (2020). The association between socioeconomic factors and weight loss 5 years after gastric bypass surgery. Int J Obes.

[33] Cadena-Obando D, Ramírez-Rentería C, Ferreira-Hermosillo A, Albarrán-Sanchez A, Sosa-Eroza E, Molina-Ayala M, Espinosa-Cárdenas E (2020). Are there really any predictive factors for a successful weight loss after bariatric surgery?. BMC Endocr Disord.

[34] Laurino Neto RM, Herbella FA, Tauil RM, Silva FS, de Lima SE Jr. (2012). Comorbidities remission after Roux-en-Y gastric bypass for morbid obesity is sustained in a long-term follow-up and correlates with weight regain. Obes Surg.

[35] Stroh CE, Nesterov G, Weiner R, Benedix F, Knoll C, Pross M, Manger T (2014). Circular versus linear versus hand-sewn gastrojejunostomy in Roux-en-Y-gastric bypass influence on weight loss and amelioration of comorbidities: data analysis from a quality assurance study of the surgical treatment of obesity in Germany. Front Surg.

[36] Edholm D (2019). Systematic review and meta-analysis of circular- and linear-stapled gastro-jejunostomy in laparoscopic Roux-en-Y gastric bypass. Obes Surg.

[37] Fehervari M, Alyaqout K, Lairy A, Khwaja H, Bonanomi G, Efthimiou E (2020). Gastrojejunal anastomotic technique. Does it matter? Weight loss and weight regain 5 years after laparoscopic Roux-en-Y gastric bypass. Obes Surg.

[38] Andreu A, Jimenez A, Vidal J, Ibarzabal A, De Hollanda A, Flores L, Cañizares S, Molero J, Moizé V (2020). Bariatric support groups predicts long-term weight loss. Obes Surg.

[39] Luca P, Nicolas C, Marina V, Sarah B, Andrea L (2021). Where are my patients? Lost and found in bariatric surgery. Obes Surg.

