# Correction to: Comparison of Linear Versus Circular-Stapled Gastroenterostomy in Roux-en-Y Gastric Bypass: a Nationwide Population-Based Cohort Study

**DOI:** 10.1007/s11695-021-05474-y

**Published:** 2021-05-14

**Authors:** Marleen M. Romeijn, Stijn van Hoef, Loes Janssen, Kelly G. H. van de Pas, François M. H. van Dielen, Arijan A. P. M. Luijten, Kevin W. A. Göttgens, Jan Willem M. Greve, Wouter K. G. Leclercq

**Affiliations:** 1 Department of Surgery, Máxima Medical Center, Veldhoven, The Netherlands; 2 Research School NUTRIM, Department of Surgery, Maastricht University Medical Center, Maastricht, the Netherlands; 3 Department of Surgery, Zuyderland Medical Center, Heerlen, the Netherlands

**Correction to: Obesity Surgery.**

10.1007/s11695-021-05436-4

In **T**able 1, the headings in the top row are missing. The names in this heading represent the two groups of the study: CSA (left column) and LSA (right column).

**Table 1:** Baseline characteristics of the study population

	**CSA,** ***n*** **= 881**	**LSA,** ***n*** **= 11,587**	***p*** **value**
Gender, no. (%)			
Female	713 (80.9)	9541 (82.3)	.291
Age (years)	45.2 ± 10.5	45.1 ± 10.6	.885
Preoperative comorbidities, no. (%)			
Hypertension Type II diabetes mellitus Hyperlipidaemia Gastroesophageal reflux disease OSAS Osteoarthritis	333 (37.8) 143 (16.2) 169 (19.2) 89 (10.1) 265 (30.1) 344 (39.0)	4269 (36.8) 2595 (22.4) 2523 (21.8) 1667 (14.4) 2231 (19.3) 5699 (49.2)	.571 <.001* .071 <.001* <.001* <.001*
Preoperative weight (kg, ±SD)	122.7 ± 18.6	122.7 ± 17.9	.994
Preoperative BMI (kg/m^2^ ± SD)	42.7 ± 4.9	42.8 ± 4.7	.265
Laparoscopic, no. (%)	879 (99.8)	11,573 (99.9)	.396
Length of biliopancreatic limb (cm ± SD)	72.9 ± 15.3	90.5 ± 38.6	<.001*
Length of alimentary limb (cm ± SD)	145.8 ± 9.7	133.6 ± 33.7	<.001*
Length of hospital stay (days ±SD)	2.3 ± 1.7	1.5 ± 2.7	<.001*
Number of readmission (<30 days), no. (%)	25 (2.8)	283 (2.4)	.466
Postoperative complication <30 days, no. (%)			
CD grade I CD grade II CD grade IIIa CD grade IIIb CD grade IVa CD grade IVb CD grade V	19 (2.2) 25 (2.8) 3 (.3) 41 (4.7) 4 (.5) - -	89 (.8) 99 (.9) 29 (.3) 382 (3.3) 15 (.1) 9 (.1) 1 (.0)	<.001* <.001* .610 .032* .017* .408 .783
Type of complication, no. (%)			
Major bleeding Anastomotic leakage Intra-abdominal abscess Wound infection Intestinal obstruction Anastomotic stricture	21 (2.4) 1 (.1) 2 (.2) 1 (.1) 1 (.1) 0 (.0)	136 (1.2) 43 (.4) 13 (.1) 14 (.1) 28 (.2) 1 (.0)	.002* .214 .343 .952 .447 .783

The corrected table is shown below.

