# Supplementary material for: Comparison of Linear versus Circular-Stapled Gastroenterostomy in Roux-en-Y Gastric Bypass: A Nationwide Population-Based Cohort Study
Source: Obes Surg. 2021 Apr 27;31(8):3579–87. doi: 10.1007/s11695-021-05436-4 (PMC8270800; doi:10.1007/s11695-021-05436-4)
Supplement: Supplementary file 1 — (DOCX 13 kb) [file 11695_2021_5436_MOESM1_ESM.docx]

|  | **CSA, n= 881** | **LSA, n= 11587** | **p-value** |
| --- | --- | --- | --- |
| *Postoperative complications* ≥30 days *, no. (%)* Marginal ulcer  Anastomotic stricture  Dumping syndrome  Gall stone formation  Incisional hernia  Bowel obstruction  Internal hernia | 2 (.2)  -  4 (.5)  27 (3.1)  7 (.8)  5 (.6)  39 (4.4) | 23 (.2)  12 (.1)  113 (1.0)  165 (1.4)  16 (.1)  12 (.1)  334 (2.9) | .855  .339  .112  <.001*  <.001*  <.001*  .009* |

**Supplementary table 1.** Long-term complications comparing circular stapled and linear stapled gastroenterostomy during RYGB

*p value is below the threshold of <.05.
Abbreviations: CSA= circular-stapled anastomosis, LSA= linear-stapled anastomosis
